# A randomized control trial: training program of university students as health promoters

**DOI:** 10.1186/1471-2458-13-162

**Published:** 2013-02-22

**Authors:** Víctor Manuel Mendoza-Núñez, Cecilia Mecalco-Herrera, Cosme Ortega-Ávila, Laura Mecalco-Herrera, Juan Luis Soto-Espinosa, Mario Alfredo Rodríguez-León

**Affiliations:** 1 Facultad de Estudios Superiores Zaragoza, Universidad Nacional Autónoma de México (UNAM), Guelatao # 66, Col. Ejército de Oriente, México, DF, 09230, Mexico

**Keywords:** University health promoter, University student health promoter, Self-care, Health promotion

## Abstract

**Background:**

Several studies have reported the following as determining factors for the adoption of healthy lifestyles among undergraduate students: gender, socioeconomic level, prior lifestyles, environment, parental lifestyles and health status, career choice, and healthy support networks. However, these factors are influenced by students’ knowledge about healthy lifestyles.

**Methods/design:**

We will carry out a randomized trial in a sample of 280 new undergraduate students at the National Autonomous University of Mexico’s Faculty of Higher Studies-Zaragoza (FES-Zaragoza, UNAM). There will be an experimental group (n = 140), comprising 20 students from each of the seven university departments (careers); these students will receive training as university student health promoters through an e-learning course. This course will allow the topics necessary for such promoters to be reviewed. There will be a control group (n = 140), comprising 20 students from each of the seven departments (careers); these students will not undergo the training. Later, the students who comply satisfactorily with the e-learning course will replicate the course to 10 of their classmates. A healthy-lifestyle questionnaire will be given to all the participants, and the parameters established in the self-care card will be recorded before and after the training. The study variables are as follows: (i) independent variable—compliance with the e-learning course; (ii) dependent variables—lifestyles changes prior to the educative intervention (including healthy eating, physical activity, and addiction prevention) and parameters related to health status established in self-care (including weight, body mass index, waist circumference, and hip circumference). Data will be analyzed using Student’s *t* test and logistic regression analysis odds ratios with 95% confidence intervals. The analysis of the open answers will be carried out with ATLAS. ti 5.5 software.

**Discussion:**

Health promotion among university students should incorporate options that are feasible for and attractive to students. Thus, as proposed in the present protocol, e-learning courses offer excellent possibilities because they allow students to program their learning in their available time without affecting their academic studies.

**Trial registration:**

http://ISRCTN77787889

## Background

Integrative professional development encompasses ethical, affective, humanistic, esthetic, sociopolitical, and health-care dimensions in addition to theoretical, disciplinary knowledge and obtaining technical competencies [1-3]. In this regard, it has been demonstrated that there is a relationship between university students’ health status and academic performance—hence; implementing health-promotion programs during their academic development is important [4]. However, it has been observed that many university students do not attach importance to their health care and even report their health status as being good or very good despite their lifestyles being unhealthy [5].

Self-care is understood as an individual’s reasoned behavior; it has a theoretical basis that allows the individual to act and decide on the prevention, diagnosis, and treatment of disease as well as maintaining their health and enjoying maximal quality of life (QOL) according to their sociocultural context. It constitutes an operative strategy of empowerment for maintaining health that is fundamental to developing all dimensions of a future professional’s integrative development [6,7].

QOL and health status differ even among countries in the same region, as has been demonstrated in European university students. In this respect, among British university students, it was observed that 70% of them did not comply with recommended physical activity, 66% consumed less than the recommended amount of fruits and vegetables, and 56% consumed alcohol at least once a week [8]. Likewise, there are great gender differences: women report less smoking, lower alcohol intake, and lower use of illegal substances, although they consume more fruits and vegetables. Males have a higher level of physical activity, consume fewer sweets, and have more restorative sleep [9]. It has also been reported that females have a lower frequency of psychological problems [10]. On the other hand, in a study carried out on Spanish students, it was found that 68.4% of males and 48.4% of females engaged in physical activity according to recommended levels. Similarly, students who undertook physical activity consumed more fruits and vegetables and smoked less than those who did not do any physical activity. In the same study, it was found that sedentary males devoted more time to using a computer, and sedentary females devoted more time to watching television [11]. The academic antecedents and lifestyles of the students’ parents were determining factors in physical exercise among the young people. The results of that studies suggest that healthy lifestyles are linked to behavior of social nets conformed, which supports the notion that health is transmittable.

Among Greek university students, it was observed that although females reported less physical exercise than males, their nutrition was better and their rates of overweight and obesity were lower than those of males. Likewise, medical students reported a lower frequency of alcohol consumption, although there were no differences with respect to eating habits compared with other university majors [12].

With respect to alcohol consumption, better habits have been observed in female Swedish university students than in males; however, females reported greater psychological stress, and males showed a higher percentage of overweight and obesity linked to refuse of advice on nutrition and worse healthy lifestyles [13]. On the other hand, Bulgarian students reported consuming sweets, cakes, and snacks more frequently, whereas Polish students consumed fewer fruits and vegetables. In general, males consumed more snacks than females (except in Bulgaria), and students who lived in home with their parents consumed more fruits and vegetables than those who lived alone or with friends [14].

On the other hand, in a study carried out among Lebanese university students, 37% overweight and 12.5% obesity was observed in males; 13.6% overweight and 3.2% obesity was recorded in females. In contrast, 6.4% of females and 1% of males were underweight [15]. Likewise, 22.9% overweight or obesity was recorded among Canadian university students; females were more concerned about their body weight than males, although their lifestyles and health status did not show significant differences [16].

In general, it has been demonstrated that women are more concerned about their body weight than men. Women exhibit better health habits, although they report more psychological problems [12-17].

One relevant aspect to student lifestyles is the changes observed with time at university, particularly if the students’ studies involve health, as in the case of medical students. In this respect, the 15% of the London students were non-drinkers, among those who drank, 48% of the men and 38% of the women exceeded sensible weekly limits of alcohol consumption [18]. Also it has been observed that the consumption of alcohol diminished with their time at university [19]. This supports the proposal of implementing a university health-promotion program so that all students—irrespective of their majors—possess adequate knowledge about adopting healthy lifestyles.

The present study will be carried out at the National Autonomous University of Mexico’s Faculty of Higher Studies-Zaragoza (FES-Zaragoza, UNAM). This university campus is a UNAM multidisciplinary faculty and was founded in 1976; currently, it offers undergraduate studies in the following seven academic majors: (i) medical surgery (*n* = 1,434); (ii) dental surgery (*n* = 1,672); (iii) nursing (*n* = 1,357); (iv) psychology (*n* = 2605); (v) biology (*n =* 1398); (vi) biological/pharmacological chemistry (*n* = 1,652); and (vii) chemical engineering (*n* = 1039). There are in addition some postgraduate courses.

In some unpublished studies conducted on students at FES- Zaragoza, UNAM, it was found that 40% of students were either overweight or obese as a consequence of unhealthy lifestyles. Surprisingly, students undertaking careers in the health sciences (medicine, nursing, odontology, and psychology) had a higher percentage of overweight and obesity than those in biological and chemical sciences (biology, chemical engineering, pharmaceutical chemistry, and biochemistry). This suggests that though their academic education is linked with health promotion, it is insufficient to change their lifestyles. It underlines the importance of complementary actions to promote the integrative development of health self-care regardless of the students’ academic discipline. Therefore, FES-Zaragoza, UNAM has implemented the University Health Promoter (UHP) model as a policy for integrative professional formation. This constitutes a fundamental strategy for training students in the adoption of healthy lifestyles during their academic development.

The UHP model incorporates health promotion as an educative work project. It has the purpose of promoting human development and improving the QOL of those who study or work. At the same time, it encourages healthy behavior in families, future work environments, and in society in general [20-23].

Among the determining factors for adopting healthy lifestyles, it is necessary to examine gender, socioeconomic level, prior lifestyles, health status, the environment, parental lifestyles and health status, career choice, healthy support networks [24], and overall students’ knowledge about strategies to adopt healthy lifestyles during their time at university. Within this framework, the aim of the present study is to assess the impact of a training program to promote health among university students. Consequently, the trained university students will help create a safe, healthy learning environment at FES-Zaragoza, UNAM, which will in turn promote the development of an integrative health culture. At the same time, healthy lifestyles in terms of self-care will be promoted in the student community.

## Methods

**Figure 1:**
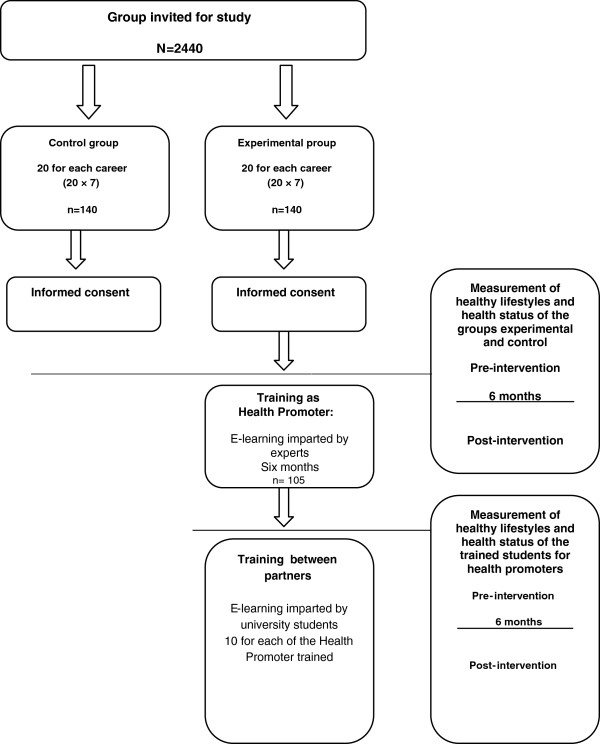
General scheme.

After receiving signed informed consent from the students for participation, we will carry out a randomized trial using a sample of 280 new undergraduate students at FES-Zaragoza, UNAM. The groups will be as follows. (i) An experimental group (n = 140) of 20 students will be made up from each of the seven departments (careers); these participants will receive training as “university student health promoters” through an e-learning course, in which the topics established for a UHP will be reviewed [21,22] (Table 1). (ii) The control group (n = 140) will consist of 20 students from each of the seven departments (careers), but they will undergo no training (Figure 1).

**Table 1:** E-learning topics for the training of university health promoters

-My virtual classroom	-Body hygiene and health
-University student health determinants	-Safe and healthy environment
-How does my body work	-Addiction prevention
-Communication for health	-Conflict management
-Student leadership and health	-Recreation and health
-Health promotion: university health promoter	-Health and cultural activities
-Self-care	-Student support networks and health self-monitoring
-Healthy eating	-First Aid
-Physical activity and health	-University tutoring for health
-Self-esteem and health	-Ethics and health self-care

### Sample size

For the calculation of sample size it was assumed a prevalence of 40% of overweight and obesity considering the results of the unpublished studies. We considered a significance level of 5%, a difference in clinical interest of 10% related to the control group, and a precision of 2.5% with a power of the 90%.

A healthy-lifestyle questionnaire will be given to all the participants, and the parameters will be detailed in the self-care card before and after the training (Additional file 1: Table S2 and Additional file 2: Table S3). The study variables will be as follows: (i) independent variable—compliance with the e-learning course for promoting health; (ii) dependent variables—lifestyles changes prior to the educative intervention (including healthy eating, physical activity, and addiction prevention) and parameters related to health status in self-care (including weight, body mass index, waist circumference, and hip circumference); and (iii) intervenient variables—age, gender, health status before training, current lifestyle (before training), family income, academic course, parents’ schooling, and parents’ health status. All dependent variables will be measured prior to and after the educative intervention.

Later, the students who comply satisfactorily with the e-learning course will replicate the course to 10 of their classmates. We are considering a follow-up every 6 and 12 months during 4 years.

Data will be processed using SPSS ver. 15.0 (SPSS, Inc., Chicago, IL, USA) statistical software. Descriptive statistics will be presented as means ± standard deviations. Results will be analyzed using Student’s *t* test and logistic regression analysis odds ratios with 95% confidence intervals. Pearson’s correlations will be obtained and *p* <0.05 will be considered significant. Analysis of the open answers will be conducted using ATLAS. ti 5.5 software.

## Discussion

FES-Zaragoza, UNAM has established the following as its Institutional Development Plan Mission: “To form undergraduate and postgraduate Health Sciences, Social Sciences, and Chemical-Biological Sciences professionals; with a multidisciplinary focus; with the skills to participate actively in the generation and updating of scientific knowledge; with institutional identity, ethics and social commitment, and capable of complementing their formation with the development of healthy lifestyles, participation in cultural activities and civic responsibility.” Thus, among its strategic objectives the following can be recognized: promoting the integrative development of students and operating the faculty as a healthy, sustainable, safe unit [25].

In some studies performed among Mexican undergraduate students, similarities have been observed with findings reported in European universities: over 25% overweight, 10% obesity, and 10% underweight [26,27]. In this regard, it has been demonstrated that a high percentage of students who are overweight or obese perceive a low QOL [28]. In addition, they suffer from eating disorders, such as bulimia and vomiting induction (emetics) for weight control [29].

With regard to the risk factors associated with the health status of Mexican university students, it has been reported that there is a much higher prevalence of overweight and obese males than females [27]. Similarly, over 50% of students reported familial antecedents of high blood pressure or type 2 diabetes mellitus [30]. Likewise, self-esteem problems have been observed with lack of communication and racism [31].

Concerning responsible sexuality, it has been reported that inconsistent use of condoms in persons with more than two sexual partners constitutes a significant risk factor for acquiring human papillomavirus (HPV) [32].

Within this context, we suppose that the main determining factors for developing self-care among FES-Zaragoza, UNAM undergraduate students will be similar to those reported in other studies, such as age, gender, health status, lifestyle, and parental schooling and lifestyle (11–18). Therefore, the implementation of significant educative programs will create a favorable environment that will exert a positive impact on the healthy behavior of students as part of their integrative development.

Health promotion among university students should incorporate options that are feasible for and attractive to students. In this respect, as proposed in the present protocol, e-learning courses offer excellent possibilities because they allow students to program their learning in their available time without affecting their studies. Similarly, the strategy whereby students trained as university health promoters pass on the knowledge they have acquired to their peers (peer training) will have a multiplying effect. It will allow the language and strategies employed by young university students themselves to exert a significant impact. This is because they will render the terms and concepts for maintaining health according to their communication codes; they will emphasize the priority aspects within a university student health-promotion network.

In addition, we consider it indispensable to cultivate a healthy university environment through the sale of healthy foods (fruits and vegetables) on campus and to provide the students with drinking water on campus. Physical activity should also be promoted during the breaks between classes. Efforts should be made to strengthen students’ self-esteem in the university health-promotion networks established by the students.

The study’s results will permit FES-Zaragoza, UNAM to achieve the premises, objectives, and goals of a health promoter university, allowing its experience to be compared with that of other universities.

Finally, we assume that the adoption of healthy lifestyles by university students will have an impact on their family and community environment, and in the case of health sciences students it will have a positive effect on their professional practice.

## Competing interests

The authors declare that they have no competing interests.

## Authors’ contributions

All the authors made an intellectual contribution to this study. VM M-N conceived the study, oversaw its design and implementation, and drafted the manuscript. C M-H participated in the study design and coordination and helped draft the manuscript. C O-A, L M-H, JL S-E, and MA R-L contributed to the program and the design of the e-learning course, self-card, and virtual classroom. All authors read and approved the final manuscript.

## Pre-publication history

The pre-publication history for this paper can be accessed here:

http://www.biomedcentral.com/1471-2458/13/162/prepub

## Supplementary Material

Additional file 1: Table S2 Healthy lifestyles-associated self-effectiveness questionnaire. Click here for file

Additional file 2: Table S3 Self-care card for university students. Click here for file
