# Supplementary material for: A randomized control trial: training program of university students as health promoters
Source: BMC Public Health. 2013 Feb 22;13:162. doi: 10.1186/1471-2458-13-162 (PMC3608970; doi:10.1186/1471-2458-13-162)
Supplement: Additional file 1: Table S2 — Healthy lifestyles-associated self-effectiveness questionnaire. [file 1471-2458-13-162-S1.doc]

**Additional file 1: Table S2. Healthy lifestyles-associated self-effectiveness questionnaire**

| **Healthy lifestyles self-report***  1. Eating  How would you score the compliance level of self-care actions for healthy eating on a scale from 1 (lowest) to 10 (highest)? Mark with an (**X**).   | 1 | 2 | 3 | 4 | 5 | 6 | 7 | 8 | 9 | 10 | | --- | --- | --- | --- | --- | --- | --- | --- | --- | --- |   Cite three strategies that you have used to comply with this measurement.  1.______________________________________________________  2.______________________________________________________  3.______________________________________________________  Cite three difficulties or problems that you have faced concerning compliance with this measurement.  1.______________________________________________________  2._______________________________________________________  3._______________________________________________________  Write three proposals for improving compliance with this measurement.  1.______________________________________________________  2.______________________________________________________  3.______________________________________________________  **__________**  ***Same format for:** (i) physical exercise, (ii) self-esteem, (iii) body hygiene, (iv) environmental hygiene, (v) sleep, (vi) ingestion of alcoholic beverages, (vii) smoking, (viii) addictions, (ix) surveillance of health status, (x) responsible sexuality, (xi) communication, (xii) conflict management, (xiii) recreation, and (xiv) cultural activities. |
| --- | --- | --- | --- | --- | --- | --- | --- | --- | --- | --- |
