# Supplementary material for: A randomized control trial: training program of university students as health promoters
Source: BMC Public Health. 2013 Feb 22;13:162. doi: 10.1186/1471-2458-13-162 (PMC3608970; doi:10.1186/1471-2458-13-162)
Supplement: Additional file 2: Table S3 — Self-care card for university students. [file 1471-2458-13-162-S2.doc]

**Additional file 2: Table S**3. Self-care card for university students

| **SELF-CARE CARD**  **Name:_________________________________________________________________________**  **Age___________ Gender_________ University career**____________________   |  | **2012**  **(I)** | **2012**  **(II)** | **2013**  **(I)** | **2013**  **(II)** | **2014**  **(I)** | **2014**  **(II)** | **2015**  **(I)** | **2015**  **(II)** | **2016**  **(I)** | **2016**  **(II)** | | --- | --- | --- | --- | --- | --- | --- | --- | --- | --- | --- | | Weight (kg) |  |  |  |  |  |  |  |  |  |  | | Height (m) |  |  |  |  |  |  |  |  |  |  | | BMI (kg/m^2^) |  |  |  |  |  |  |  |  |  |  | | Waist circumference (cm) |  |  |  |  |  |  |  |  |  |  | | Hip circumference (cm) |  |  |  |  |  |  |  |  |  |  | | WHI |  |  |  |  |  |  |  |  |  |  | | Cardiac frequency at rest |  |  |  |  |  |  |  |  |  |  | | Respiratory frequency at rest |  |  |  |  |  |  |  |  |  |  | | BP (systolic/diastolic, mm/Hg) |  |  |  |  |  |  |  |  |  |  | | Smoking (average amount of cigarettes smoked daily) |  |  |  |  |  |  |  |  |  |  | | Alcoholic beverages (average drinks or beers ingested weekly) |  |  |  |  |  |  |  |  |  |  | | DMF index |  |  |  |  |  |  |  |  |  |  | | PDI |  |  |  |  |  |  |  |  |  |  | | Hemoglobin |  |  |  |  |  |  |  |  |  |  | | Hematocrit |  |  |  |  |  |  |  |  |  |  | | Glucose |  |  |  |  |  |  |  |  |  |  | | Cholesterol |  |  |  |  |  |  |  |  |  |  | | HDL |  |  |  |  |  |  |  |  |  |  | | LDL |  |  |  |  |  |  |  |  |  |  | | Triglycerides |  |  |  |  |  |  |  |  |  |  | | Self-esteem scale (Rosenberg SES) |  |  |  |  |  |  |  |  |  |  | | Depression scale (Zung SDS) |  |  |  |  |  |  |  |  |  |  | | WHOQOL-BREF |  |  |  |  |  |  |  |  |  |  |   **The registry will be carried out every 6 months (in February and August) except for shadowed parameters, which will be measured yearly (in February). BMI, Body mass index; WHI, Waist-hip index; BP, Blood pressure; DMF, Decayed/Missing/Filled teeth index; PDI, Periodontal disease index; HDL, High-density lipoproteins; LDL, Low-density lipoproteins; Zung (SDS), Zung self-rating depression scale; RSES, Rosenberg self-esteem scale; WHOQOL-BREF, World Health Organization Quality of Life-Brief Version.** |
| --- | --- | --- | --- | --- | --- | --- | --- | --- | --- | --- | --- | --- | --- | --- | --- | --- | --- | --- | --- | --- | --- | --- | --- | --- | --- | --- | --- | --- | --- | --- | --- | --- | --- | --- | --- | --- | --- | --- | --- | --- | --- | --- | --- | --- | --- | --- | --- | --- | --- | --- | --- | --- | --- | --- | --- | --- | --- | --- | --- | --- | --- | --- | --- | --- | --- | --- | --- | --- | --- | --- | --- | --- | --- | --- | --- | --- | --- | --- | --- | --- | --- | --- | --- | --- | --- | --- | --- | --- | --- | --- | --- | --- | --- | --- | --- | --- | --- | --- | --- | --- | --- | --- | --- | --- | --- | --- | --- | --- | --- | --- | --- | --- | --- | --- | --- | --- | --- | --- | --- | --- | --- | --- | --- | --- | --- | --- | --- | --- | --- | --- | --- | --- | --- | --- | --- | --- | --- | --- | --- | --- | --- | --- | --- | --- | --- | --- | --- | --- | --- | --- | --- | --- | --- | --- | --- | --- | --- | --- | --- | --- | --- | --- | --- | --- | --- | --- | --- | --- | --- | --- | --- | --- | --- | --- | --- | --- | --- | --- | --- | --- | --- | --- | --- | --- | --- | --- | --- | --- | --- | --- | --- | --- | --- | --- | --- | --- | --- | --- | --- | --- | --- | --- | --- | --- | --- | --- | --- | --- | --- | --- | --- | --- | --- | --- | --- | --- | --- | --- | --- | --- | --- | --- | --- | --- | --- | --- | --- | --- | --- | --- | --- | --- | --- | --- | --- | --- | --- | --- | --- | --- | --- | --- | --- | --- | --- | --- | --- | --- | --- | --- | --- | --- | --- | --- | --- | --- | --- | --- | --- | --- | --- | --- | --- | --- |
